# Designing a Finite Element Model to Determine the Different Fixation Positions of Tracheal Catheters in the Oral Cavity for Minimizing the Risk of Oral Mucosal Pressure Injury: Comparison Study

**DOI:** 10.2196/69298

**Published:** 2025-07-11

**Authors:** Zhiwei Wang, Zhenghui Dong, Xiaoyan He, ZhenZhen Tao, Jinfang QI, Yatian Zhang, Xian Ma

**Affiliations:** 1 The Sixth Affiliated Hospital of Xinjiang Medical University, No. 39 Wuxing South Road, Tianshan DistrictUrumqi, Xinjiang, 830000, China, 86 15999102329; 2 Department of Critical Care Medicine, Qinghai Fifth People's Hospital, Xining, China; 3 Emergency Department, The Fourth Affiliated Hospital of Xinjiang Medical University, Xin Jiang, China; 4 School of Nursing, Xinjiang Medical University, Xin Jiang, China

**Keywords:** tracheal catheter, fixed position, oral mucosal pressure injury, finite element, biomechanical analysis

## Abstract

**Background:**

Despite being an important life-saving medical device to ensure smooth breathing in critically ill patients, the tracheal tube causes damage to the oral mucosa of patients during use, which increases not only the pain but also the risk of infection.

**Objective:**

This study aimed to establish finite element models for different fixation positions of tracheal catheters in the oral cavity to identify the optimal fixation position that minimizes the risk of oral mucosal pressure injury.

**Methods:**

Computed tomography data of the head and face from healthy male subjects were selected, and a 3D finite element model was created using Mimics 21 and Geomagic Wrap 2021 software. A pressure sensor was used to measure the actual pressure exerted by the oral soft tissue on the upper and lower lips, as well as the left and right mouth corners of the tracheal catheter. The generated model was imported into Ansys Workbench 22.0 software, where all materials were assigned appropriate values, and boundary conditions were established. Vertical loads of 2.6 N and 3.43 N were applied to the upper and lower lips, while horizontal loads of 1.76 N and 1.82 N were applied to the left and right corners of the mouth, respectively, to observe the stress distribution characteristics of the skin, mucosa, and muscle tissue in four fixation areas.

**Results:**

The mean (SD) equivalent stress and shear stress of the skin and mucosal tissues were the lowest in the left mouth corner (28.42 [0.65] kPa and 6.58 [0.16] kPa, respectively) and progressively increased in the right mouth corner (30.72 [0.98] kPa and 7.05 [0.32] kPa, respectively), upper lip (35.20 [0.99] kPa and 7.70 [0.17] kPa, respectively), and lower lip (41.79 [0.48] kPa and 10.02 [0.44] kPa, respectively; *P*<.001 for both stresses). The equivalent stress and shear stress of the muscle tissue were the lowest in the right mouth angle (34.35 [0.52] kPa and 5.69 [0.29] kPa, respectively) and progressively increased in the left mouth corner (35.64 [1.18] kPa and 5.74 [0.30] kPa, respectively), upper lip (43.17 [0.58] kPa and 8.91 [0.55] kPa, respectively), and lower lip (43.17 [0.58] kPa and 11.96 [0.50] kPa, respectively; *P*<.001 for both stresses). The equivalent stress and shear stress of muscle tissues were significantly greater than those of skin and mucosal tissues in the four fixed positions, and the difference was statistically significant (*P*<.05).

**Conclusions:**

Fixation of the tracheal catheter at the left and right oral corners results in the lowest equivalent and shear stresses, while the lower lip exhibited the highest stresses. We recommend minimizing the contact time and area of the lower lip during tracheal catheter fixation, and to alternately replace the contact area at the left and right oral corners to prevent oral mucosal pressure injuries.

## Introduction

The primary method of respiratory support for critically ill patients in the intensive care unit (ICU) is oral tube intubation, which ensures airway patency, increases ventilation volume, and enhances lung function. However, the use of oral tube intubation may lead to oral mucosal pressure injury (OMPI) due to excessive or prolonged pressure, friction, and shear forces [1]. OMPI can increase patient pain, elevate the risk of infection, impose a financial burden on health care, increase staff workload, and even result in medical disputes. The incidence of OMPI in patients in the ICU ranges from 2.95% to 49.2%, with different fixation positions and methods of tracheal catheterization influencing its occurrence [2]. While numerous factors contribute to OMPI, including patient-related factors, physiological conditions, the use of specific medications, and nursing-related aspects, there are limited reports addressing the mechanical factors that cause OMPI [3-5]. The International Guidelines for the Clinical Prevention and Treatment of Stress Injuries suggest that finite element models can be employed to evaluate mechanical factors by assessing stress distribution characteristics within tissue structures and predicting the risk of cellular and tissue damage [6].

The purposes of this study were to use the finite element theory contact algorithm to simulate and analyze the compression process of the oral soft tissue when the endotracheal tube is fixed in different fixed positions in the oral cavity, and to explore the stress distribution characteristics of the oral soft tissue under the force of the endotracheal tube. This would help to more realistically and accurately evaluate the actual force on the oral soft tissue structure and to clarify the reasonable fixed position of the endotracheal tube when it is fixed in the oral cavity, so as to prevent the occurrence of OMPI.

## Methods

### Finite Element Model

A finite element model of the tracheal catheter positioned at various locations within the mouth was established. The selected participant for the head and facial computed tomography scan was a 28-year-old male volunteer with a normal BMI, measuring 175 cm in height and weighing 72 kg. A total of 512 images, each with a thickness of 0.625 mm, were obtained. The DICOM format data were imported into the 3D reconstruction software Mimics (version 21.0; Materialise) and Geomagic Wrap (version 2021; Raindrop) for model fitting and structural segmentation, respectively. A resistive film pressure sensor was employed to measure the actual pressure exerted by the tracheal catheter in different areas of the patient’s mouth, with each measurement being repeated 100 times to calculate an average value using the gravitational formula. Subsequently, using the measured pressures from solid models as the input data, the Ansys software (version 22.0; ANSYS) was used to import the optimized model, define material properties, remesh the model, and generate an accurate finite element model to conduct finite element analysis based on the defined elastic modulus, Poisson ratio, boundary conditions, and simulated loads for various tissues (skin mucosa and muscle tissue), as well as the tracheal catheter and bone [7,8]. The properties of each material are shown in Table 1; the skin and mucosa are set as nonlinear materials, and the bones are set as isotropic materials

**Table 1.** Material properties of the finite element model.

Material	Modulus of elasticity (Mpa）	Young modulus (Mpa）	Shear modulus (Mpa）	Poisson ratio (%）
Tracheal catheter	3	—^a^	1500	0.38
Skeleton	13,400	18,000	—	0.25
Muscle	0.045	0.25	—	0.49
Cutaneous mucosa	—	3	2	0.49

a not available.

### Ethical Considerations

This study was approved by the Ethics Committee of the Sixth Affiliated Hospital of Xinjiang Medical University (approval number: LFYLLSC20220905-01). All procedures in this study are in line with the ethical standards of the Human Experiments Responsible Committee (Institution and State) and the Declaration of Helsinki.

### Setting of Boundary Conditions

In this study, four models representing the upper lip, lower lip, left mouth corner, and right mouth corner were established. The fixed support areas of the models were designated as the top and bottom, allowing for rigid support to be simulated through fixed constraints. A sliding friction contact was implemented between the lip and the tracheal tube, with a friction coefficient set at 1 [9]. A bonded connection was established among the skin, mucous membrane, and muscle tissue. The model accounted for the effects of gravity in a vertical downward direction, with a gravitational acceleration of 9.8 m/s².

### Measurement Indicators

The equivalent stress and shear stress of the skin mucosa and muscle tissue were measured under different fixed positions of the tracheal catheter within the mouth. The stress distribution characteristics of the pressure injury model were analyzed for the fixed positions of the upper lip, lower lip, left mouth corner, and right mouth corner. The stress measurement for each part was conducted 10 times to obtain an average value.

### Statistical Analysis

Statistical analysis was performed using SPSS (version 25.0; IBM Corp). Measurement data were expressed as mean (SD). One-way ANOVA was employed for comparisons between groups, while the *t* test was used for intragroup comparisons. A *P* value of less than .05 was considered statistically significant.

## Results

### Model Verification

A finite element model of the tracheal catheter was established with a total of 14,635 nodes and 8267 elements at various fixed positions within the oral cavity. This model included the ilium of the upper and lower jaws, as well as the skin, mucosa, and muscle tissues of the oral cavity. The extreme values and distribution trends of stress at the mouth angle and lower lip were consistent with the findings of Amrani et al [9], indicating the effectiveness of the modeling approach employed in this study.

### Equivalent Stress

The equivalent stress of the skin mucosa was the lowest in the left mouth corner, and then progressively increased in the right mouth corner, upper lip, and lower lip. In contrast, the equivalent stress of muscle tissue was the highest in the right mouth corner, followed by the left mouth corner, upper lip, and lower lip. Notably, the equivalent stress of muscle tissue was significantly greater than that of the skin mucosal tissue (*P*<.001; Table 2).

**Table 2.** Comparison of equivalent stress results between skin mucosa and muscle tissue (kPa, n=10).

Position	Cutaneous mucosa, mean (SD)	Muscle tissue, mean (SD)	*t* test *(df)*	*P* value	95% CI
Upper lip	35.20 (0.99)	43.59 (0.84)	−20.371 (9)	<.001	−9.252 to −7.522
Lower lip	41.82 (0.92)	48.35 (0.92)	−15.927 (9)	<.001	−7.389 to −5.667
Left mouth corner	28.42 (0.65)	35.64 (1.18)	−16.924 (9)	<.001	−8.118 to −6.325
Right mouth corner	30.72 (0.99)	34.34 (0.38)	−10.789 (9)	<.001	−3.420 to −2.912
*F*_1_-score	430.942	573.406	N/A^a^	N/A	N/A
*P* value	<.001	<.001	N/A	N/A	N/A

a not available.

### Shear Stress

The shear stress of the skin mucosal tissue was the lowest in the left mouth corner, and progressively increased in the right mouth corner, upper lip, and lower lip. In contrast, the shear stress of the muscle tissue was the lowest in the right mouth corner, and progressively increased in the left mouth corner, upper lip, and lower lip. At the four fixed positions, the shear stress of the left and right oral muscle tissue was lower than that of the skin mucosa, while the shear stress of the upper and lower lip muscle tissue was higher than that of the skin mucosal tissue (*P*<.005; Table 3)

**Table 3.** Comparison of shear stress results between the skin mucosa and muscle tissue (kPa, n=10).

Position	Cutaneous mucosa, mean (SD)	Muscle tissue, mean (SD)	*t* test *(df)*	*P* value	95% CI
Upper lip	7.60 (0.21)	8.91 (0.39)	−8.959 (9)	<.001	−1.613 to −0.998
Lower lip	10.17 (0.16)	11.69 (0.78)	−5.057 (9)	<.001	−2.145 to −0.882
Left mouth corner	6.58 (0.17)	5.79 (0.33)	6.799 (9)	.001	0.543 to 1.030
Right mouth corner	7.45 (0.36)	5.69 (0.29)	11.972 (9)	<.001	1.450 to 2.068
*F_1_*-score	244.363	126.411	N/A^a^	N/A	N/A
*P* value	<.001	<.001	N/A	N/A	N/A

a not available.

### Comparison of Equivalent Stress and Shear Stress in the Mucosal Tissue of the Upper and Lower Lips and the Left and Right Mouth Corners

Equivalent stress was found to be lower in the upper lip compared to the lower lip, and the left mouth corner exhibited lower stress than the right mouth corner (*P*<.001; Table 4-5). In terms of shear stress, the upper lip also showed significantly lower values than the lower lip (*P*<.001;Table5), while the left mouth corner had lower shear stress than the right mouth corner (*P*<.001; Table 5).

**Table 4.** Comparison of the results of equivalent stress and shear force in the left and right mouth corners (kPa, n=10).

Position	Left side mouth corner, mean (SD)	Right side mouth corner, mean (SD)	*t* test (*df*)	*P* value	95% CI
Equivalent stress	28.42 (0.65)	30.72 (0.99)	−6.160 (9)	<.001	−3.094 to −1.520
Shear stress	6.58 (0.17)	7.45 (0.36)	−6.984 (9)	<.001	−1.125 to −0.605

**Table 5.** Comparison of the results of equivalent stress and shear force in the skin mucosal tissue of the upper and lower lip (kPa, n=10).

Position	Upper lip, mean (SD)	Lower lip, mean (SD)	*t* test *(df)*	*P* value	95% CI
Equivalent stress	35.20 (0.99)	41.82 (0.92)	−15.472 (9)	<.001	−7.519 to 5.721
Shear stress	7.60 (0.21)	10.17 (0.16)	−16.769 (9)	<.001	−2.931 to −2.279

### Comparison of Equivalent Stress and Shear Stress in the Muscle Tissue of the Upper and Lower Lips and Left and Right Mouth Corners

The equivalent stress was the lower in the upper lip than in the lower lip (*P*<.001), and higher in the left mouth corner than in the right mouth corner (*P*=.004; Table 6). The shear stress was lower in the upper lip than in the lower lip (*P*<.001), and lower in the left mouth angle than in the right mouth angle (*P*=.298; Table 7)

**Table 6.** Comparison of equivalent stress and shear force results in the left and right mouth corners (kPa, n=10).

Position	Left side mouth corner, mean (SD)	Right side mouth corner, mean (SD)	*t* test (*df*)	*P* value	95% CI
Equivalent stress	35.64 (1.18)	34.34 (0.38)	3.308 (9)	.004	0.474 to 2.124
Shear stress	5.74 (0.30)	5.69 (0.29)	1.071 (9)	.50	−0.221 to 0.435

**Table 7.** Comparison of equivalent stress and shear force results in the muscle tissue of the upper and lower lips (kPa, n=10).

Position	Upper lip, mean (SD)	Lower lip, mean (SD)	*t* test *(df)*	*P* value	95% CI
Equivalent stress	43.59 (0.84)	48.35 (0.92)	−12.115 (9)	<.001	−5.587 to −3.935
Shear stress	8.91 (0.39)	11.69 (0.78)	−12.477 (9)	<.001	−3.561 to −2.545

### Stress Distribution Rules of the Four Groups of Models

The equivalent stress range of the skin mucosa and muscle tissue gradually extends from the stress center to the periphery. In this study, the application direction of the forces on the upper and lower lips is vertical, with the maximum peak values of both equivalent stress and shear stress occurring at the stress point and subsequently radiating outward in the vertical direction. Conversely, the forces applied at the left and right mouth corners are horizontal, causing the stress range to spread horizontally, with the highest stress values appearing at the direct contact point between the tracheal catheter and the mucosal tissue. The distribution of shear stress is centered on the soft tissue stress point and encompasses the entire lip, mandibular region, and both sides of the face, resulting in a broader range of stress. The equivalent stress and shear stress at the mouth corners are significantly lower than those at the upper and lower lips.

**Figure 1.**
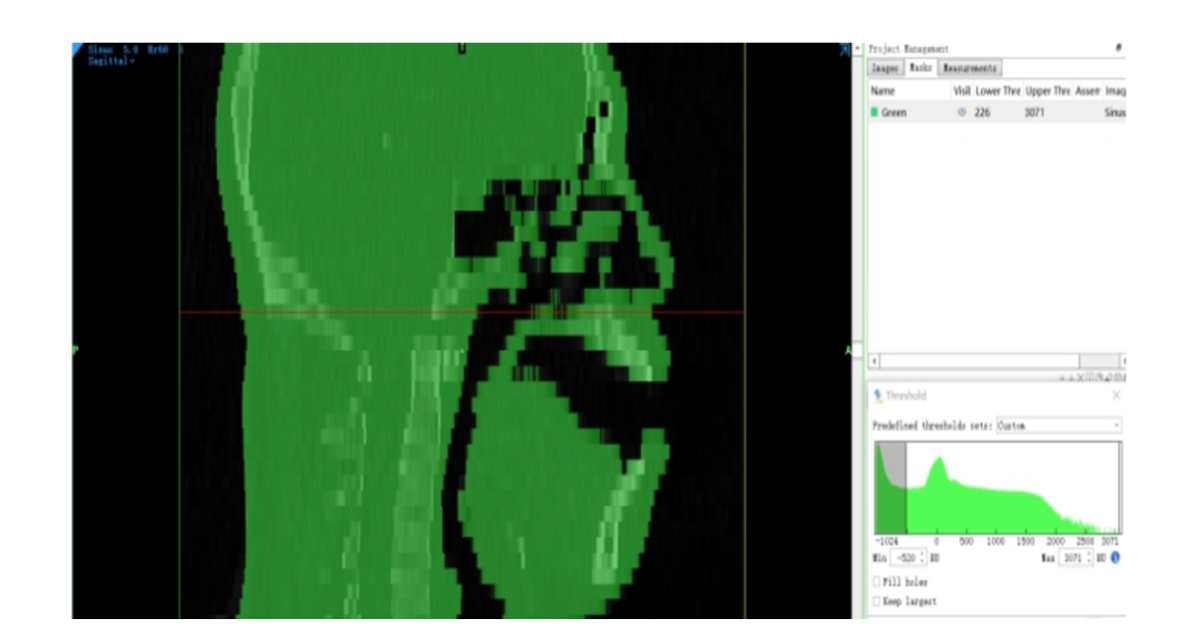
Mimics21.0 software was used to reconstruct the patient's head, face and oral tissues in 3D with an interval of 0.25 mm, and the contour range of the skin mucosa and muscle tissue was constructed through the thresholds of different tissues.

To explore the underlying reasons, when the tracheal catheter is fixed at the corner of the mouth, it makes contact with the corner, the upper lip, and the lower lip. The pressure, shear force, and friction generated by this contact are dispersed across the three contact surfaces of the mouth and the upper and lower lips. The contact surface between the tracheal tube and the upper and lower lips serves as the primary stress point, leading to greater stress values at the upper and lower lips compared to the corners of the mouth, with the lower lip experiencing the highest stress. The results of the finite element analysis indicate that the stress at the corners of the mouth is lower, followed by that at the upper lip (Figures1^4^).

**Figure 2.**
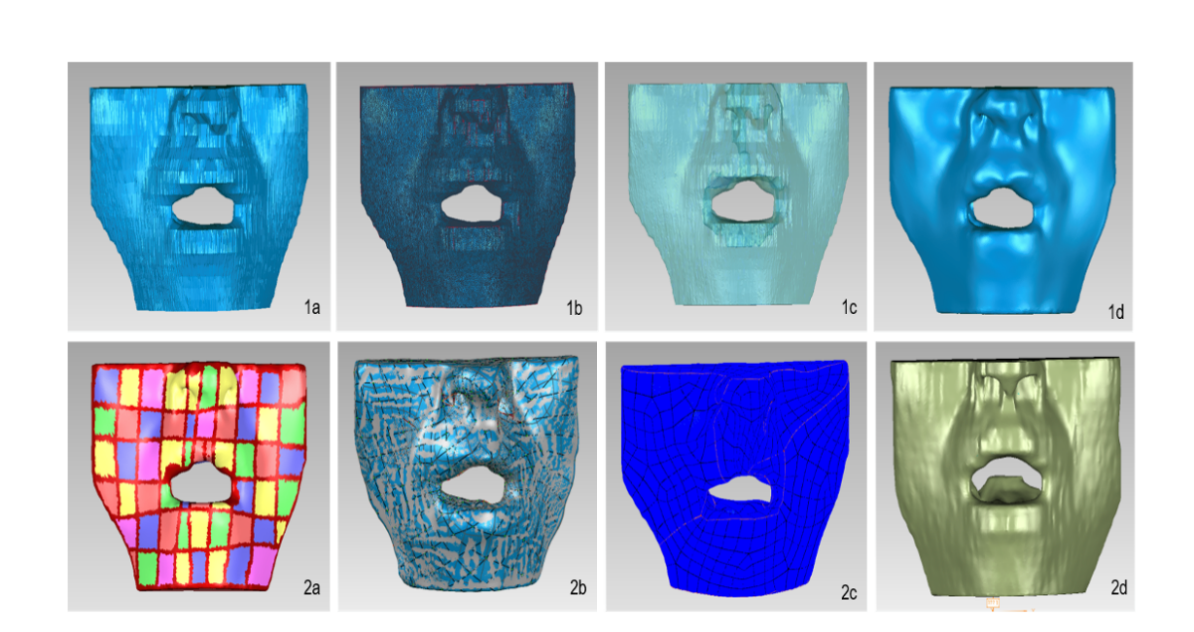
The probe contour line is used to redraw the contour line of the model, so that the surface pieces are more extensible and the concave and convex surfaces are reduced. The structural patch trims the model patch again to make the patch smoother and smoother, which is consistent with the characteristics of the skin tissue. Construct grids, and optimize and adjust all patch nodes and elements. Finally, the fitting surface constructs a model that is similar to the actual oral and facial features of the human body.

**Figure 3.**
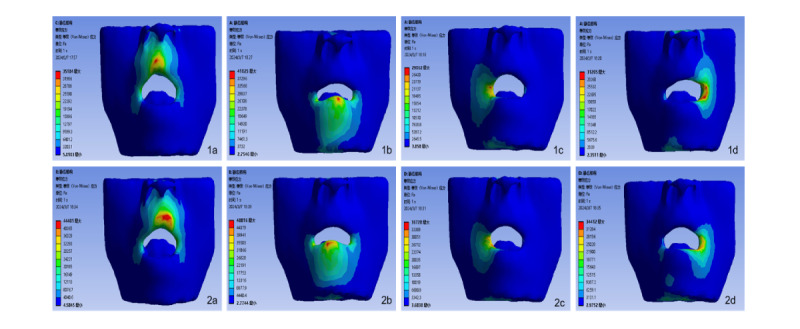
(1a-1d) Equivalent stress nephogram of two tissues at 4 fixed locations: upper lip, lower lip, left mouth corner, and right mouth corner. (2a-2d) Equivalent stress nephogram of the muscle tissue of the upper lip, lower lip, left mouth corner, and right mouth corner.

**Figure 4.**
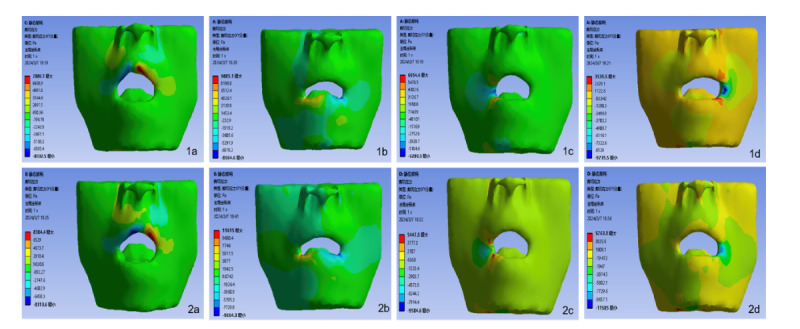
Shear stress nephogram of two tissues at 4 fixed locations. (1a-1d) Shear stress nephogram of the mucosa tissue of the upper lip, lower lip, left mouth corner, and right mouth corner. (2a-2d) Shear stress nephogram of the muscle tissue of the upper lip, lower lip, left mouth corner, and right mouth corner.

## Discussion

The results of this study showed that when the tracheal tube was in contact with the lower lip, the equivalent stress and shear stress values of muscle tissue and mucosal tissue were the largest, followed by the upper lip, and the left and right mouth angles were lower than those of the upper and lower lip. Finite element analysis modeling is a powerful bioengineering technique employed to assess tissue loading, encompassing the interactions between tissues, objects, and medical devices. This numerical method effectively addresses mechanical problems [10]. It enables rapid and accurate stress-strain analysis of the structure, shape, load, and mechanical properties of materials in any given model [11]. Moreover, finite element analysis objectively and accurately reflects the distribution of stress, strain, and deformation, and has gained widespread application in oral biomechanics research in recent years [12].

The tracheal catheter is a critical instrument for mechanical ventilator-assisted therapy in patients in the ICU; however, the catheter itself and improper fixation methods may lead to OMPI [6]. From a biomechanical perspective, the OMPI associated with tracheal catheters primarily results from vertical pressure, shear forces, and friction [13]. Continuous mechanical loading on soft tissues is the main contributor to stress injuries, typically occurring at bony prominences or in areas contacting medical devices. When skin or deep tissue deformation persists for a certain duration owing to the pressure from medical devices, pressure injuries may develop [14]. In this study, the mechanical load originated from the force exerted by the tracheal catheter on the oral soft tissue. Contact between the tracheal catheter and the oral mucosal tissue resulted in continuous pressure, leading to tissue deformation in the mucosa. Research indicates that tracheal catheters and their fixation devices are stiffer than oral soft tissues. When the mechanical properties of these instruments do not align with those of the soft tissues, deformation occurs in the latter, concentrating mechanical stress and strain at the points of direct contact, which then gradually extends to the surrounding areas [15,16].

Continuous vertical pressure on soft tissues is a significant factor in the occurrence of stress injuries. The incidence of OMPI correlates with the intensity and duration of pressure; the greater the pressure and the longer its application, the higher the risk of developing OMPI is [17]. Furthermore, when the tracheal tube is improperly fitted and fixed too tightly, the pressure and shear force exerted will increase [14]. Shear forces applied to deep skin tissues can obstruct capillaries, leading to localized ischemia and hypoxia, which may result in deep tissue necrosis. Consequently, damage from shear forces is often undetected in the early stages and is more challenging to heal than damage from typical wounds [13]. Friction arises from the movement between the oral mucosal tissue and the surface of the tracheal tube; while it does not directly cause OMPI, it can compromise the epidermal cuticle, leading to the shedding of the mucosal surface layer and heightened sensitivity to pressure injuries. Once the compromised oral mucosal tissue is subjected to stimuli from saliva and other secretions, the risk of pressure injury escalates. Additionally, friction raises the temperature of the local mucosal tissue, disrupts the local microenvironment, alters pH levels, and increases tissue oxygen consumption, further exacerbating tissue ischemia and heightening the risk of OMPI [16].

The magnitude of the internal mechanical load required to cause tissue damage depends on the duration of the applied force and the specific biomechanical tolerance of the stressed tissue, which is influenced by factors such as age, shape, health status, and the functional capacity of the body systems, including tissue repair ability [18]. Both high loads applied for short durations and low loads sustained over extended periods can lead to tissue damage [18-20]. Continuous loading is one of the primary contributors to this damage; it refers to loads applied over prolonged periods (ranging from a few minutes to several hours or even days), also known as quasi-static mechanical loading. Research indicates that when soft tissues come into contact with the support surfaces of medical devices, pressure and shear forces are generated between the soft tissues and these surfaces [21]. This interaction results in distortion and deformation of the soft tissues under pressure, affecting both the skin and deeper tissues (including fat, connective tissue, and muscle), leading to stress and strain within the tissues [21]. Excessive internal stress in the tissues can disrupt intracellular material transport by damaging cellular structures (such as the cytoskeleton or plasma membrane) or by hindering the transport process itself (for example, by reducing blood perfusion, impairing lymphatic function, and affecting material transport in the interstitial space), which can ultimately result in cell death and trigger an inflammatory response. Concurrently, the emergence of endothelial cell spacing increases vascular permeability, leading to inflammatory edema, which further exacerbates the mechanical load on cells and tissues due to elevated tissue pressure, thus contributing to the development of pressure injuries [22-24].

According to the results of finite element analysis, the stress experienced by the lower lip is the highest, followed by the upper lip, with levels significantly exceeding those at the corners of the mouth. Therefore, in clinical practice, when fixing a tracheal catheter, it is advisable to select the mouth corner to maximize the contact surface area between the catheter and this region. Placing the tracheal catheter in the middle of the mouth minimizes the contact time between the catheter and the oral mucosa. Additionally, regular changes in the fixation position can help redistribute pressure, thereby reducing pressure, shear forces, and friction on the oral mucosa, ultimately lowering the risk of OMPI.

This study analyzed alterations in the stress experienced by oral soft tissue under pressure at various fixation positions of the tracheal catheter within the mouth, from a biomechanical perspective. It provides a theoretical foundation for preventing OMPI in patients with tracheal catheters in the ICU. While this study effectively simulates the biomechanical effects of contact between oral soft tissue and the tracheal catheter, it does not fully replicate the actual forces experienced by oral soft tissue in real-life situations, as the area of contact between the tracheal catheter and the oral soft tissue cannot be completely simulated. Additionally, the study included only one young adult male, which limits the generalizability of the findings. Therefore, it is essential to include participants of varying genders and ages to enhance the scientific validity of the research. Furthermore, improvements in the identification rate and curvature of the 3D grid of the model should be pursued to generate higher-quality 3D models, thereby enhancing data accuracy.

## Abbreviations

ICU: intensivec are unit
OMPI: oral mucosal pressure injury
